# Machine learning in mental health and its relationship with epidemiological practice

**DOI:** 10.3389/fpsyt.2024.1347100

**Published:** 2024-03-11

**Authors:** Marcos DelPozo-Banos, Robert Stewart, Ann John

**Affiliations:** ^1^ Swansea University Medical School, Swansea, United Kingdom; ^2^ King’s College London, Institute of Psychiatry, Psychology and Neuroscience, London, United Kingdom; ^3^ South London and Maudsley National Health Service (NHS) Foundation Trust, London, United Kingdom

**Keywords:** mental health, epidemiology, machine learning, research methods, challenges and opportunities

## Introduction

It is fair to say that the application of machine learning (ML) in healthcare has not been smooth. The field of ML has let down the medical community and the wider public in many respects: from research that is clinically irrelevant (1) or applying flawed methodologies (2), to non-transparent sharing of data with industry (3, 4). Success stories do exist across a range of physical health specialties, but they currently remain a minority (5, 6).

In hindsight, the pitfalls for ML in medical research are hardly surprising. Epidemiology (medicine’s own approach) is underpinned by statistics and hypothesis testing, designed to maintain ethical etiquette, ensure robust, unbiased results, and produce strong evidence and knowledge in measured phenomena – at least in principle (7). It therefore aims to understand the ‘true’ mechanisms connecting exposures and outcomes (features and targets in ML jargon) and naturally gravitates towards simpler, easier to interpret models. ML has its own established methodology (8), but one that is fundamentally different, geared towards solving problems and developing applications (9). It therefore pursues maximum accuracy at predicting the outcome and naturally prefers complex, more powerful models. The different use of logistic regression by both fields illustrates this. While epidemiology takes special care with correlated independent variables and directs its attention to the estimated coefficients, ML mostly disregards these and focusses on predictive power. Overall, while both epidemiology and ML rely on data to obtain their results, their core principles are at odds. Nevertheless, appropriately introducing ML elements into epidemiological research is possible and guidelines have been published (10).

**Figure 1:**
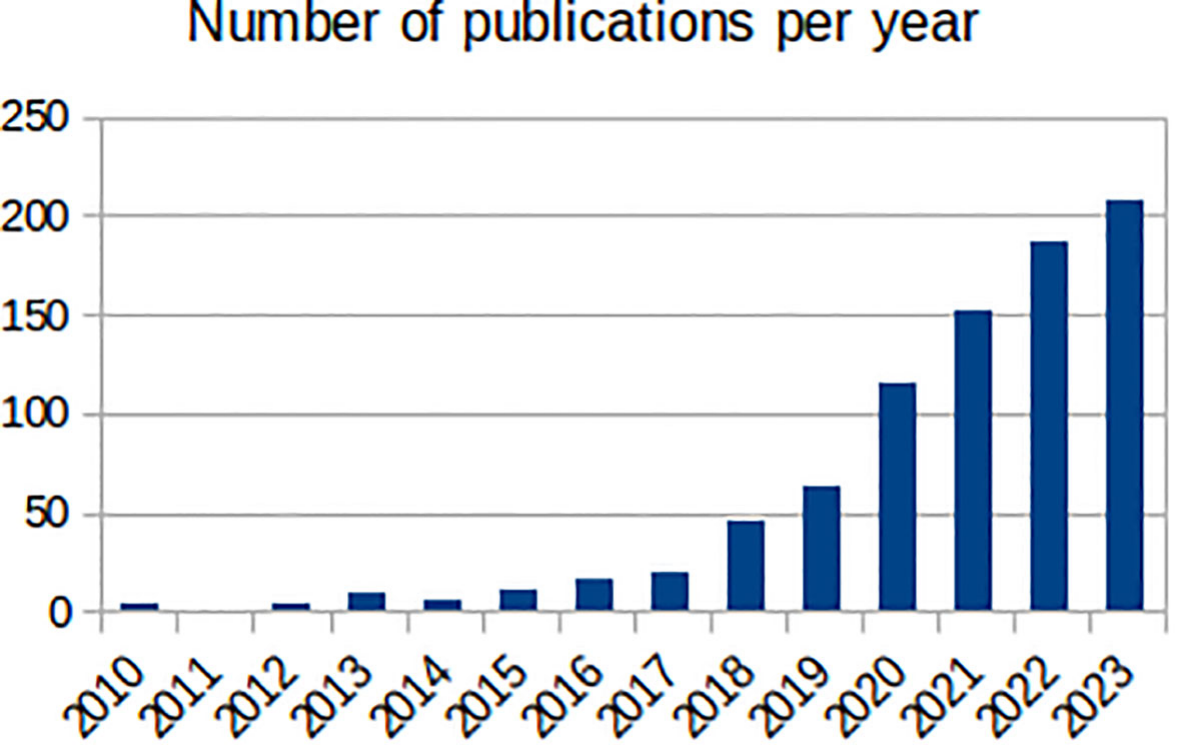
Number of annual publications found by PubMed (https://pubmed.ncbi.nlm.nih.gov/) between, 2010 and, 2023 with the terms “machine learning” and one of “mental health”, “mental illness”, “depression”, “anxiety”, “bipolar disorder, “schizophrenia”, “psychotic disorder”, “ADHD” or “autism” in the title.

**Figure 2:**
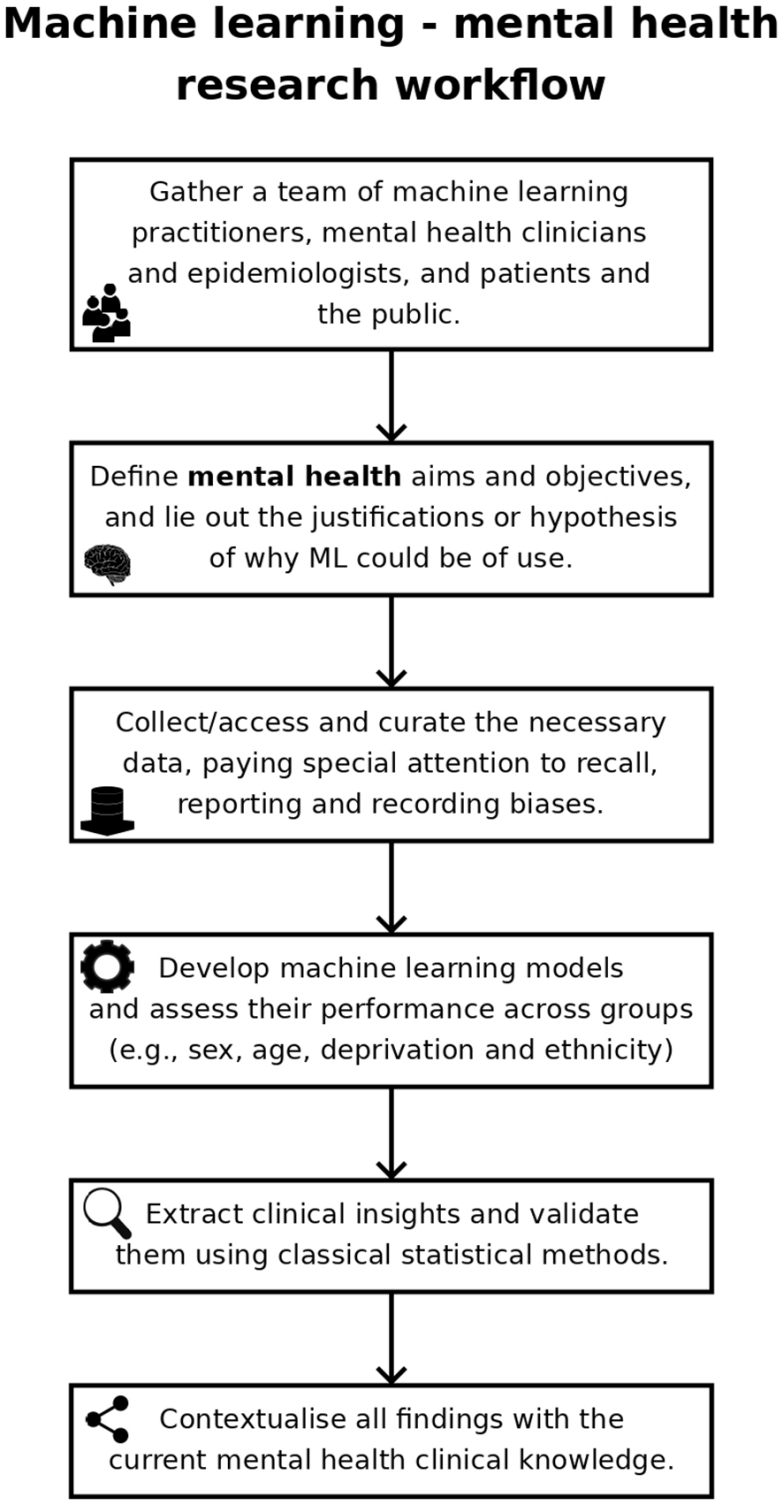
Typical workflow of machine learning – mental health research.

Mental health has been a target for ML, with the number of ML mental health publications increasing dramatically since, 2017 (**Figure 1**), and the research community is rightly expectant of its impact. However, the challenges are amplified: (1) losing sight of mental health objectives, over-promising on data processing and problem-solving (9); (2) technical hurdles of multiple underlying biases and often heightened privacy requirements (11); and (3) difficulties building, validating and approving ML-enabled clinical devices for diseases with insufficiently clear underlying mechanisms (12). Overall, it is the responsibility of individual researchers and institutions alike to demonstrate the value of ML for mental health. Here, we reflect on these ideas and their corresponding steps within the workflow of ML mental health research (**Figure 2**), in the hope of bringing awareness to the field and to elicit further conversations.

## The ideal target for ML

Factors affecting an individual’s mental health extend far beyond the clinical setting and are numerous, with complex interactions. Social, demographic, and economic factors and people’s psychological make-up carry as much or more weight in estimating risk of mental health outcomes as medical symptoms, biological factors, and previous health (e.g., the effect of loneliness in suicidal thoughts and self-harm) (13). The complexity of these relationships is typified in suicide research, where a meta-analysis of risk factors identified little progress in prevention over a span of 50 years (14). Consequently, the heavy reliance of classical statistics on prior expert knowledge and model assumptions is another important limiting factor in mental health research. Progress in data provision and data linkage has addressed some of the challenges of mental health research (i.e., providing better population coverage and a wider range of risk factors) but has brought additional challenges such as larger volumes of data, lower data quality, increased missing data and unstandardised phenotypes (15, 16). Furthermore, the field of mental health is evolving, and expert consensus is lacking on the taxonomy of psychiatric diagnosis (17) or on preferred ‘transdiagnostic’ clinical phenotypes (18).

The complexity and wide reach of its disease models are why mental health might particularly benefit from ML. ML is better equipped than classical statistics to deal with large numbers of factors, complex (i.e., non-linear) interactions, and noise (i.e., low quality or missing data, unstandardised phenotypes) (19). A data-driven approach is of particular value (20), such as deep learning techniques (21), and ML could be pivotal in evidence provision for diagnostic taxonomies or clinical phenotypes. However, this requires demonstrable evidence on applied clinical validity.

## Keeping sight of mental health aims and objectives

Single studies of ML predicting an outcome from a given dataset, and therefore only presenting performance results of these models, are of limited interest for mental health research (22). More valuable applications seek to improve our understanding of the disease (e.g., risk factors or time trends) and/or identify intervention opportunities. Therefore, researchers working on ML mental health should strive to: (1) extract new clinical insights from their models; (2) validate such insights with supplementary statistical analyses, and (3) contextualise their findings in the existing clinical literature. Completing all three objectives in full is not always possible, but researchers should make an honest effort on each of them and, when unsuccessful, acknowledge it as a limitation of their research.

This is not to say that research aiming at developing new ML algorithms and methodologies to process data with similar characteristics to those from mental health data (outlined below) are unimportant. Such research may naturally rely on mental health data, but the focus is on the fundamental characteristics of the data, not its mental health content – indeed, the research could have been completed using any other (non-mental health) data with the same fundamental characteristics. In this scenario, researchers should recognise that their work is about ML and not mental health, and this should be reflected in the focus of their papers and their targeted audience.

## ML challenges when using mental health data

Data curation is a critical part in developing ML models for healthcare. Some of the steps involved in this process are identical to those seen in epidemiological research: determining the sample size through power calculations; assessing the quality of the variables; studying bias in the patterns of missing data and recording practices; and evaluating the representation of the study population by the study sample. Other data curation steps are more specific to ML: the need for larger volumes of data, especially for complex models (23); comprehensive evaluation of outcome variable quality (24); data partition strategies for model building and validation (in ML jargon *training* and *testing*; *cross-validation*, often done repeatedly to improve robustness and generalizability of the results) (25); and considering additional security measures to prevent data inference from the ML model itself (in ML jargon *membership inference attacks*) (26).

Many of the data curation steps described above are potentially more complex in mental health research. Recall and reporting biases are common in self-reported mental health data, and can lead to under- or overestimation of underlying associations (27). When these biases affect the outcome variable, the entire validity of the model can be compromised. With ML being a “data driven” approach, these biases can be especially damaging in ML applications. They should therefore be reduced as much as possible to improve the model’s performance and clinical validity, with the remaining bias carefully considered when assessing the results (24). Additionally, achieving participation and retention of participants in mental health research may also be challenging (28). The use of routinely collected electronic health records alleviates these issues to some extent; however, many important constructs of interest are subjective and can only be self-reported. Furthermore, minority groups (often the most affected by mental health inequalities) and those with more severe syndromes are frequently excluded and underserved (29). In addition, outcomes such as self-harm are known to be under-recorded in electronic health records (30). More generally, mental health data are viewed as relatively sensitive, partly due to the personal nature of the questions asked in a typical clinical assessment but also due to the stigma surrounding mental health conditions and consequent heightened privacy concerns – the public is slightly less inclined to share their mental health data for research compared to their physical health (31). This results in additional ethical and legal hurdles for mental health research (32), and more so for the application of ML due to its need of large volumes of data and the risk of models inadvertently carrying these data (26).

There is no *easy fix* for these problems, and, compared to most other medical specialties, mental health researchers, especially those applying ML, often need to: (1) focus more resources on their data curation strategy; (2) address bias in their data with statistical tools such as inverse probability weighing, which can be applied to both epidemiology (33) and ML (34) methods; and (3) have a stronger patient and public involvement and engagement plan (35).

## ML enabled clinical mental health devices with unknowns

The path from the lab to the clinical setting for medical innovations is not simple. This is especially true for ML-enabled devices, and still under discussion (5, 36) with regulatory frameworks evolving (37). In fact, only a small proportion of the published clinical ML research has been focused on deployment (5); as of October 19, 2023, the United States Food and Drug Agency reports approving less than 700 ML-enabled medical devices (based on their summary descriptions) (38), although this is likely an underestimation due to bias in explicit reporting of ML methods (39).

The situation is exacerbated for clinical mental health devices, with less real-world deployments (40) and fewer FDA approved devices (6). This may reflect the currently restricted scope of such devices as a consequence of our limited knowledge of the mechanisms underlying mental disorders, at least relative to other specialties (12). Without such knowledge, ML models are often fed a wide range of risk factors suspected to be related to the outcome (or in the hope that they will be of value during prediction). The assumption here is that if a model accurately predicts the outcome, it must be a true representation of the real-world phenomena described by the data. However, the data may contain variables that are confounders or act as proxies to latent variables, thus rendering the assumption unfair. When the *potential* risk factors fed to the ML algorithm lack evidence supporting and explaining their relationship with the outcome (as it is often the case), the clinical validity of the resulting ML-enabled mental health device remains to be proven, regardless of its accuracy. However, with the clinical knowledge laid down, healthcare professionals and patients will be more likely to accept the *black box* quality of ML models (41), and ML will have a clearer path to developing mental health solutions.

## Individual and collective responsibility

Researchers have a responsibility to demonstrate that, when correctly applied, ML can lead to improved knowledge and care of mental health disorders. To achieve this, ML practitioners must work in close collaboration with mental health epidemiologists and clinicians, and actively seek their input to protocol design and data interpretation. Crucially, they need to acknowledge that data fed into ML models represent personal experiences, to be aware of the particular sensitivities of mental health data, and to learn to handle these data responsibly above and beyond legislated privacy and security requirements. Conversely, mental health researchers seeking to engage with ML must avoid being blinded by the hype. Instead, they must continue to adhere to the main methodological principles of epidemiology and mental health research, and scrutinise any ML models generated (42). They should also be cautious of utilizing easy-to-use ML libraries and tools without the appropriate training, as these have led to the abuse and misuse of ML by non-experts (43).

Organisations and large projects could play a key role in ensuring that the fields of mental health and ML interact as described here. For example, DATAMIND (the MRC funded, UK Hub for Mental Health Data Science; www.datamind.org.uk) brings the issues outlined above to the attention of the field of mental health research at large, holding regular meetings and conferences with a wide range of stakeholders, and providing mental health data science workshops for early career researchers. DATAMIND is also developing a set of standardised mental health phenotypes to be used by the scientific community (44) and contributing to the cataloguing of available mental health data resources to improve discoverability and accessibility (45). Crucially, DATAMIND achieves this in close collaboration with academics, healthcare professionals, industry, and, most importantly, patients and people with lived experiences.

## Concluding remarks

Overall, the opportunity of using ML in mental health is not cost-free. As described, it introduces complexity, especially in mental health research, and additional workflow steps. Therefore, its application in healthcare generally, and in mental health particularly, needs to be justified. Ideally, this should be done at the planning stage, evidencing why the use of ML is needed to solve an existing problem that is hindering research: for example, to reduce an original set of available measurements to a size that is more manageable for traditional statistical regression (46). Alternatively, the benefits of using ML over conventional statistical methods can be treated as a hypothesis to be tested as part of the research project: for example, by comparing how well ML and statistical models fit the used data.

Beyond the hype, ML can genuinely play a central role in the future of psychiatry and mental healthcare. However, this depends on researchers applying ML responsibly and avoiding the mistakes seen in its application to other medical specialties.

## Author contributions

MDPB: Writing – review & editing, Writing – original draft, Conceptualization. RS: Writing – review & editing. AJ: Writing – review & editing.
